# Social impact of behavioral disorders in psychiatric patients

**Published:** 2012

**Authors:** M Ladea, MC Şinca, D Ferechide, LS Grosu

**Affiliations:** * “Prof. Dr. Alexandru Obregia” Clinical Hospital of Psychiatry, Bucharest; ** Specialty Doctor in Psychiatry, Bucharest; *** “Carol Davila” University of Medicine and Pharmacy, Bucharest

**Keywords:** mental health disorders, violence, offenders

## Abstract

**Background:** Violent behavior is often met in patients with mental health disorders. An important area of research studied different factors that can influence aggressive behavior in psychiatric patients.

**Objectives:** The paper’s aim was to compare different characteristics between two groups of patients, who presented with psychiatric disorders and aggressive behavior. Demographic characteristics, diagnosis, use of alcohol, history of brain injury, physical and verbal aggression were analyzed in both groups.

**Methods:** The first group was formed of 23 mentally ill patients framed into the Article 114 Criminal Code who presented aggressive behavior and committed various offences. In the second group, 45 patients admitted at psychiatry without their consent were included, after having committed different acts of aggression.

**Results**: The patients from the first group had significantly more psychiatric admissions in their history than the patients from the second group. A higher percentage of alcohol users was registered in the first group, compared to the second one. More patients with personality disorders and concomitant use of alcohol were present in the first group, compared to the second group. In both groups, aggressive behavior was more frequent in patients having psychotic disorders, compared to other diagnosis. Even if overall aggression was more frequent in the second group, when alcohol use (with or without brain injury) was present, aggressive behavior became more frequent in the first group.

**Conclusions**: Results of the study suggest that when certain conditions are met, they can significantly influence the behavior of psychiatric patients, with notable differences in each group.

## Introduction

One of the main areas of research in psychiatry is violent and aggressive behavior in patients presenting with different mental health disorders.

Even though many individuals with psychiatric disorders do not commit violent acts, research suggests that a subset of people with psychiatric disorders present a high risk of committing assaults and violent crimes. Different observations have been inconsistent regarding the share mental illnesses contribute to this behavior and the share substance abuse, brain injury and other factors do.

In various cases, mental disorders and violent behaviors are often met at the same time in some individuals. There could be multiple factors that influence the behavior of individuals or offenders who present with mental disorders, admitted in psychiatric hospitals. Among the contributing factors for aggression and violent behavior are the following: present mental state, diagnosis, past psychiatric history, personality traits, use of alcohol and a history of brain injury.

Some theories suggest that the use of alcohol can trigger violent behavior in people with or without psychiatric disorders because these substances simultaneously impair judgment, change a person's emotional balance, and remove cognitive inhibitions [**1**].

Aggressive behaviors and violence occur in all clinical diagnostic categories. However, certain subgroups of psychiatric diagnosis have been linked with violent behavior such as personality disorders, psychotic disorders, and substance abuse disorders.

In clinical practice, assessments of the dangerousness or violence in an individual are usually based mostly on clinical judgment. Many studies which tried to assess violent behavior in mentally ill patients agreed that these patients could present higher chances to become violent than the general population, but what has remained unclear is the extent of this greater risk and how much it is modifiable or preventable [**2**].

Over the years, important researches observed different links between mental disorders and violence [**2-6**]:

1) Severe mental illness is not always a robust predictor of future violence.
2) Co-morbid substance use is vital in predicting violence.
3) People with co-occurring severe mental illness and substance abuse/dependence have a higher incidence of violence than people with substance abuse/dependence alone.
4) People with personality disorders and psychotic disorders are more prone to violent behavior than patients with affective disorders.

Other studies observed that there is also an increased risk of violence among mentally ill people with a history of violent behavior, substance abuse, brain injury and non-compliance with medications, when compared with the general population [**7**].

Research suggested that adequate treatment of mental illness and substance abuse might help reduce rates of violence. Important studies found that most patients with schizophrenia who took antipsychotics as prescribed were less likely to be violent than those who did not. Similar studies observed that patients with abuse of alcohol are less likely to adhere to treatment for a concomitant mental illness, and that can worsen psychiatric symptoms and aggressive behavior [**1**].

## Objectives

One of this paper’s aims is to compare the characteristics of two groups of patients with psychiatric disorders and aggressive behavior. Correlation between diagnosis, alcohol misuse, brain injury, physical and verbal aggression were analyzed both in patients from each group and between the two groups of patients.

We also assessed aggressive behavior and violence in different subgroups of patients depending on their diagnosis, in order to find out which subgroup of patients with mental illness carry more risk of violence than others.

## Methods

In the first group, we included 23 mentally ill patients (21 male and 2 female) who presented aggressive behavior and committed one or more offences and who came under the ambit of the law and were assessed by forensic psychiatric commission. These patients were admitted in psychiatry units during the criminal justice investigations. At the end of the investigations, all patients were framed into the Article 114 Criminal Code and the forensic psychiatric commission decided the admission in forensic psychiatric units for these patients.

The most frequent indexes of offence committed by the patients from this group were attempted murder, murder, destruction, violence, threat, attempted rape, robbery, theft and deprivation of liberty.

The second group consisted of 45 patients (39 male and 6 female) admitted without their consent in a psychiatric unit, presenting various mental health disorders, after committed different acts of aggression.

## Results

The demographical characteristics of both groups can be observed in **Table 1**.

**Table 1.** Demographical data in both groups of patients

	**First group**		**Second group**	
**GENDER**	**Frequency**	**Percent**	**Frequency**	**Percent**
Male	21	91%	39	86,67%
Female	2	9%	6	13,33%
**Total**	**23**	**100%**	**45**	**100,00%**
**AGE: mean**	**45,60 (min 18-max 70)**		**37,33 (min 18- max 64)**	
**EDUCATION**	**Frequency**	**Percent**	**Frequency**	**Percent**
Inferior	7	30,43%	14	31,11%
Medium	12	52,17%	24	53,33%
Superior	4	17,39%	7	15,56%
**Total**	**23**	**100,00%**	**45**	**100,00%**
**LIVING CONDITION**	**Frequency**	**Percent**	**Frequency**	**Percent**
With family	13	56,52%	37	82,22%
Alone	6	26,09%	8	17,78%
Not available	4	17,39%	-	-
**Total**	**23**	**100,00%**	**45**	**100,00%**
**MARITAL STATUS**	**Frequency**	**Percent**	**Frequency**	**Percent**
Married	4	17,39%	10	22,22%
Unmarried	17	73,91%	30	66,67%
Divorced	2	8,70%	5	11,11%
**Total**	**23**	**100,00%**	**45**	**100,00%**

Due to the specific of the psychiatric unit where the most part of the observation took place, the majority of the patients were male, approximately 90%, in each of the two groups.

As observed in the table above, the mean age of 45,60 in the first group was superior to the one in the second group, which was of 37,33.

In terms of education, the patients were divided into three categories: inferior (under 8 years of education), medium (between 10 and 12 years of education) and superior (university degree).

No significant differences concerning the level of education were observed in the two groups: in the first group 30,43% of patients had inferior education, 52,17% medium and 17,39% superior education; the percentage was very similar in the second group, where 31,11% of patients had less than 8 years of education, 53,33% had between 10 and 12 years, and 15,56% of the patients had university degree.

The living condition (living with family or alone) and marital status in both groups can also be observed in **Table 1**. In the second group, 82,22% of the patients were living with their family, compared to only 56,52% of those in the first group, who had the same living condition. In both groups, the majority of the patients were unmarried: 73,91% in the first group and 66,67% in the second one.

**Table 2.** Use of alcohol

**ALCOHOL USE**	**First group**		**Second group**	
	**Frequency**	**Percent**	**Frequency**	**Percent**
**No**	12	52,17%	29	64,44%
**Yes**	11	47,83%	16	35,56%
**Total**	23	100,00%	45	100,00%

**Table 3.** History of brain injury

**BRAIN INJURY**	**First group**		**Second group**	
	**Frequency**	**Percent**	**Frequency**	**Percent**
**No**	19	82,61%	44	97,78%
**Yes**	4	17,39%	1	2,22%
**Total**	23	100,00%	45	100,00%

As observed in **Tables 2** **and** **3**, in the first group, there were 47,83% of the patients with concomitant use of alcohol and 17,39% with brain injury in their medical history, compared with the second group, where 35,56% presented use of alcohol and only 2,22% history of brain injury. Results showed a higher percentage of patients with alcohol use and brain injury in the first group, compared to the second one.

**Table 4.** First contact with psychiatry

**First contact with psychiatry**	**Min**	**Mean**	**Max**
First group	1 year ago	9,9 years ago	30 years ago
Second group	1 year ago	5,85 years ago	41 years ago

The minimum period since the first contact with psychiatry was of 1 year in both groups, while the maximum period was much longer in the second group (41 years), compared to the first one (30 years).

**Table 5.** Number of previous admissions at psychiatry

**Number of admissions**	**First group**		**Second group**	
	**Frequency**	**Percent**	**Frequency**	**Percent**
Less than 3	7	30,43%	28	62,22%
Between 3 and 10	2	8,70%	11	24,44%
More than 10	14	60,87%	6	13,33%
**TOTAL**	23	100,00%	45	100,00%

**Table 5** shows the number of previous psychiatric admissions, before the one due to the offence or aggressive behavior. Significant differences can be observed between both groups: 62,22% of the patients from the second group had less than 3 previous admissions, while only 30,43% of patients from the first group had less than 3 admissions. On the other hand, the majority from the first group (60,87% of the patients) presented more than 10 previous admissions, compared to the second group, where only 13,33% had over 10 psychiatric admissions.

**Table 6.** Psychiatric disorders at the moment of the offence/aggression

**Psychiatric disorder at the moment of the offence**	**First group**		**Second group**	
	**Frequency**	**Percent**	**Frequency**	**Percent**
Affective disorder	4	17,39%	4	8,89%
Personality disorder	2	8,70%	9	20,00%
Personality disorder, use of alcohol	4	17,39%	6	13,33%
Psychotic disorder	13	56,52%	26	57,78%
**TOTAL**	23	100,00%	45	100,00%

Another important aspect was the diagnosis of the patients when admitted after committing the offence or aggression. Similar percentage of patients from each group (56,52% in the first group versus 57,78% in the second group) presented psychotic disorders (schizophrenia, acute psychotic episode and schizoaffective disorder). Personality disorder without use of alcohol was present in 8,70% of patients from the first group and 20,00% in the second group, while 17,39% of patient from the first group had personality disorder and concomitant use of alcohol, versus 13,33%, in the second group.

The data showed a higher percent of patients with personality disorder and concomitant use of alcohol in the group of patients who committed offences (first group), compared to the group of patients with aggressive behavior only (second group).

**Table 7.** Ability to discern in the group of offenders

**ABILITY TO DISCERN**	**Frequency**	**Percent**
**Absent**	16	69,57%
**Diminished**	7	30,43%
**Total**	23	100,00%

The ability to discern for every patient from the first group who committed one or more offences was established by the forensic psychiatric commission. According to the forensic psychiatric expertise, the majority of patients (69,57%) had absent ability to discern, and only 30,43% of them presented diminished ability.

**Table 8.** Aggression in both groups of patients

**Physical/verbal aggression**	**First group**		**Second group**	
	**Frequency**	**Percent**	**Frequency**	**Percent**
Physical aggression	20	86,96%	44	97,78%
Verbal aggression	9	39,13%	24	53,00%

As seen in the **Table 8**, in the first group, 86,96% of the patients presented physical aggression (N=20), compared to only 39,13% (N=9) who had verbal aggression. A high percentage of patients from the second group also presented physical aggression (97,78%), and 53,00% verbal aggression. In both groups of patients, physical aggression was clearly much more frequent than verbal aggression and overall aggression was more frequent in the second group, compared to the first one.

**Table 9.** Physical aggression in patients with alcohol use with or without brain injury

**PHYSICAL AGGRESSION**	**First group**		**Second group**	
	Frequency	Percent	Frequency	Percent
**Alcohol use**	6	30,00%	15	34,09%
**Brain injury**	0	0,00%	0	0,00%
**Alcohol use and brain injury**	4	20,00%	1	2,27%
**Total**	10	50,00%	16	36,36%

The analysis of the **physical aggression** correlated with the use of alcohol and history of brain injury showed the following results: among the patients with physical aggression from the first group (86,96%), 30,00% (N=6) presented only use of alcohol and 20,00% (N=4) of the patients had both conditions. In the second group, which had 97,78% of the patients with physical aggression, 34,09% (N=15) had only use of alcohol and 2,27% (N=1) presented both conditions.

**Table 10.** Verbal aggression in patients with alcohol use with or without brain injury

**VERBAL AGGRESSION**	**First group**		**Second group**	
	Frequency	Percent	Frequency	Percent
**Alcohol use**	4	44,44%	7	29,16%
**Brain injury**	0	0,00%	0	0,00%
**Alcohol use and brain injury**	0	0,00%	1	4,16%
**Total**	4	44,44%	8	33,32%

The same type of analysis was made in both groups for **verbal aggression,** and the outcome was the following: in the first group 39,13% presented verbal aggression, among them 44,44% (N=4) of patients had only use of alcohol and there was no patient with brain injury or both conditions simultaneously. In the second group, there were 53,00% of patients with verbal aggression, with 29,16% (N=7) having use of alcohol without brain injury and 4,16% (N=1) presented both conditions.

Both physical and verbal aggression proved to be more frequent in the second group of patients, compared to the first one, and physical aggression was more important than verbal aggression in each group of patients.

Even if, at first look, overall aggression was more important in the second group compared to the first one, it seemed that the presence of alcohol use with or without brain injury lead to a switching of the frequency rapport of overall aggression between those two groups, aggression becoming more frequent in patients included in the first group.

**Tables 11a and 11b.** Aggressive behavior and psychiatric disorder in each group

**First Group**	**Aggressive behavior**			
**Psychiatric disorder at the moment of the offence**	**Verbal aggression (N)**	**Physical aggression (N)**	**Verbal and Physical (N)**	**Total (N)**
**Affective disorder**	**2**	**1**	**1**	**4**
**Personality disorder**	**0**	**2**	**0**	**2**
**Personality disorder, use of alcohol**	**0**	**3**	**1**	**4**
**Psychotic disorder**	**1**	**8**	**4**	**13**
**TOTAL**	**3**	**14**	**6**	**23**

**Second Group**	**Aggressive behavior**			
**Psychiatric disorder at the moment of the offence**	**Verbal aggression (N)**	**Physical aggression (N)**	**Verbal and Physical (N)**	**Total (N)**
**Affective disorder**	**0**	**1**	**3**	**4**
**Personality disorder**	**1**	**4**	**4**	**9**
**Personality disorder, use of alcohol**	**0**	**3**	**3**	**6**
**Psychotic disorder**	**0**	**13**	**13**	**26**
**TOTAL**	**1**	**21**	**23**	**45**

An analysis of the aggressive behavior was performed in each group of patients, depending on their diagnosis. Results showed that in both groups the number of patients with aggressive behavior was superior in psychotic disorder sub-category, compared to the rest of them.

In both groups was also observed a similarity of distribution of the type of the aggression, depending on the diagnosis at the moment of the offence or aggression (**Tables 11a and 11b**).

**Table 12.** Personality traits in patients with physical aggression

**PHYSICAL AGGRESSION**	**First group**		**Second group**	
	**Frequency**	**Percent**	**Frequency**	**Percent**
Emotional instability/impulsivity	11	55,00%	18	40,90%
Antisocial	1	5,00%	3	6,81%
Emotional instability/impulsivity and antisocial	4	20,00%	0	0,00%
Total	16	80,00%	21	47,71%

Borderline personality disorder and antisocial personality disorder often manifest in aggression or violence **(Harvard Mental Health Letter, 2011).**

Patients from each group who presented physical aggression were analyzed after emotional instability/impulsivity and antisocial personality traits.

The results showed a higher percentage of patients with emotional instability/impulsivity (55,00%) and both antisocial and instability/impulsivity personality traits (20,00%) in patients from the first group, when compared to the second group, where only 40,90% of patients had instability/impulsivity personality traits.

Overall the presence of borderline and antisocial personality traits was more important in the group of patients who committed different offences (80,00%), compared to the group with various acts of aggression (47,71%).

## Discussion

Various researches have tried over the time to observe aggressive behavior and to predict violence in mentally ill patients.

Most studies support that mental illness does moderately increase the risk of violence. The psychiatric diagnosis, as well as psychiatric and medical history, use of alcohol, certain personality traits and other factors may contribute in making people with mental illness even more vulnerable to respond in a violent way.

Our study showed that the majority of the patients who committed offences (first group) had more than 10 previous psychiatric admissions, while the majority of the patients with acts of aggression and violent behavior (the second group) presented up to 3 previous admissions in psychiatry.

In both groups, the majority of the patients were unmarried, but while most of the patients from the second group were living with their family, just a half from the first one had the same living condition. No significant differences of years of education were observed between the two groups.

A higher percentage of patients with alcohol use was registered in the first group, compared to the second one. The number of patients with aggressive behavior was superior in psychotic disorder sub-category, compared to the rest of sub-categories.

A bigger percent of patients with personality disorder and concomitant use of alcohol was observed in the first group, compared to the second group of patients.

The presence of borderline and antisocial personality personality traits was more important in the group of patients who committed different offences, compared to the group with various acts of aggression.

## Conclusions

Even if overall aggression proved to be more frequent in the second group, when alcohol use with or without brain injury are present, aggressive behavior was observed to be more frequent in the first group.

The results of this study suggest that the use of alcohol with or without a history of brain injury may represent a contributing factor that leads to aggressive behavior, which can also influence psychiatric patients in committing offences and coming under the ambit of the law.
